# The relevance of ancient knowledge in light of contemporary insights

**DOI:** 10.4103/0019-5545.58901

**Published:** 2010

**Authors:** Shubh Mohan Singh

**Affiliations:** Department of Psychiatry, Gyan Sagar Medical College and Hospital, Banur, Punjab, India. E-mail: chotu66@rediffmail.com

Sir,

The knowledge of the ancient concepts regarding the nature of the mind are increasingly being taken note of in contemporary psychiatry.[1,2] In light of this, the article by C. Shamasundar[3] on ancient Indian wisdom and its relevance to modern mental health is timely. I believe this article also has implications for the philosophical foundations of present-day psychiatry.

Each school of philosophy is based on certain principles that differentiate it from other schools. Though cross-pollination between different schools is often desirable and productive, it is sometimes not desirable or possible to extrapolate directly from one to the other simply because they are not the same. In this regard, I would like to comment on some of the examples and conclusions that have been drawn by the author of the article.

The mind, as contemporary psychiatrists and other bio-medical professionals understand it, is a collective term for aspects of the mental state that are functions of the brain[4] and are manifested as combinations of thought, perception, memory, emotion, motivation, imagination and consciousness. These concepts are important in the study of the mind because they are easily definable, and more importantly are available for objective scrutiny and can be measured with relative ease. The author asserts that the mind is a subject of ‘academic apartheid’. It must be understood that the mind is a complex manifestation of different interlocking processes as has been pointed out above and it is not possible to talk about the mind as a unity without difficulty. Owing to this complexity, the study of the mind necessarily has to be broken down into components to make it simpler. It is expected that enquiry into the nature of these components can lead us to ‘bits’ of truth that can then be put together to get the ‘whole’ truth. As has been pointed out by the proponents of the ‘Decade of the Mind’ project,[5,6] any such study would have to focus on four broad intertwined areas of mental health, higher cognitive functions, education and computational applications. Today the study of the mind is the study of the components of the mind and numerous examples can easily be found in any neuroscience journal. The notion of ‘academic apartheid’ is difficult to maintain in this light. What is becoming increasingly uncommon is research into the nature of the mind from the point of view of thinkers such as Freud. Concepts such as the structural theory of the mind have been highly influential in psychiatry for many decades and have led to the development of many insights, but zeal for these should be tempered by the knowledge of fundamentals of science. Science is the effort to discover and increase human knowledge of the basis of the physical world by means of observation, experimentation and interpretation of the results of the former. These models of the mind have lost some of their influence because of the fact that they simply could not stand up to scientific scrutiny in any replicable manner.[4] One could argue that psychiatric nosological systems today are not valid[7] but they have undeniable usefulness as regards etiological assumptions, course and outcome and treatment implications of the nosological entities that has led us to practice psychiatry the way we do today.

The author has also described the attributes of the mind as per the different literary sources. While it cannot be denied that these concepts are valid from the points of view of the systems from which they are drawn, drawing an analogy with the aspects of the biomedical construct of the mind is difficult. I would particularly mention the examples of subliminal stimulation, mind-mind interactions, mind-body interactions and reincarnation that have been cited. These phenomena are not accepted as within the fringe of science and as per contemporary knowledge cannot be taken as scientific fact. No one can have any objections to unfettered and unconventional thinking as a way to advancement of science. Indeed many important discoveries have been made as a result of serendipity and out-of-the-box thinking, but these have entered mainstream science only after validation by a scientific method. Indeed, the quest for proof is as old as civilization itself[8] and claims such as those cited should come with a caveat about their current status. This also leads us to the question of what should be included in psychiatry and what should not. While it is difficult to exclude subjectivity from what a psychiatrist regards as the truth, it is important to be able to make a reasonable distinction between knowledge that can guide our actions and knowledge that can enable us to be aware of a contrary point of view.

The author makes a distinction between the ‘material’ and the ‘immaterial mind’ and claims that the latter cannot be recorded. Before recording the material or the immaterial mind, it is important to ask whether such a distinction exists or is indeed even desirable. The most striking example of such a distinction was propounded by Descartes who held that the mind is a non-physical substance endowed with self-awareness and consciousness that is distinct from the brain which is the seat of all intellect. This concept of dualism and its corollary that the brain is not the seat of the mind has had deleterious effects on psychiatry and the management of the mentally ill.[9,10] Contemporary psychiatry would be best served by the model proposed by Kandel[4] because it does away with all etiological assumptions except that the mind is a function of the brain and that epiphenomenonalism is false.

I would congratulate the writer for his deep knowledge lucid exposition of body-mind relationships and definition of mental health as per Indian systems of thought regarding the nature of the mind and the body. Indeed, such concepts are likely to be useful in the psychotherapeutic management of selected patients.
